# Interfaces in Epitaxially Grown Zn_3_P_2_ Nanowires and Their Composition-Dependent Optoelectronic Properties for Photovoltaic Applications

**DOI:** 10.1021/acs.chemmater.5c00985

**Published:** 2025-07-21

**Authors:** Simon Escobar Steinvall, Francesco Salutari, Jonas Johansson, Ishika Das, Sebastian Lehmann, Stephen A. Church, Maria Chiara Spadaro, Patrick Parkinson, Jordi Arbiol, Kimberly A. Dick

**Affiliations:** † Center for Analysis and Synthesis and NanoLund, 5193 Lund University, Box 124, 221 00 Lund, Sweden; ‡ Catalan Institute of Nanoscience and Nanotechnology (ICN2), CSIC and BIST, Campus UAB, Bellaterra, 08193 Barcelona, Catalonia, Spain; § Division of Solid State Physics and NanoLund, Lund University, 221 00 Lund, Sweden; ∥ Department of Physics and Astronomy and The Photon Science Institute, 5292 The University of Manchester, Manchester M13 9PL, U.K.; ⊥ Department of Physics and Astronomy “Ettore Majorana”, University of Catania and CNR-IMM, Via S. Sofia 64, 95123 Catania, Italy; # ICREA, Pg. Lluís Companys 23, 08010 Barcelona, Catalonia, Spain

## Abstract

Epitaxially grown nanowires have shown promise for photovoltaic applications due to their nanophotonic properties. Moreover, the mechanical properties of nanowires can reduce crystallographic defect formation at interfaces to help enable new material combinations for photovoltaics. One material that stands to benefit from the nanowire morphology is zinc phosphide (Zn_3_P_2_), which, despite promising optoelectronic properties, has experienced limited applicability due to challenges achieving heteroepitaxy, stemming from its incompatible lattice parameter and coefficient of thermal expansion. Herein, we identify the requirements for successful epitaxy of Zn_3_P_2_ nanowires using metalorganic chemical vapor deposition and the impact on interface structure and defect formation. Furthermore, using high-throughput optical spectroscopy, we were able to demonstrate shifts in the photoluminescence intensity and energy by tuning the V/II ratio during growth, highlighting the compositional tunability of the optoelectronic properties of Zn_3_P_2_ nanowires.

## Graphical abstract

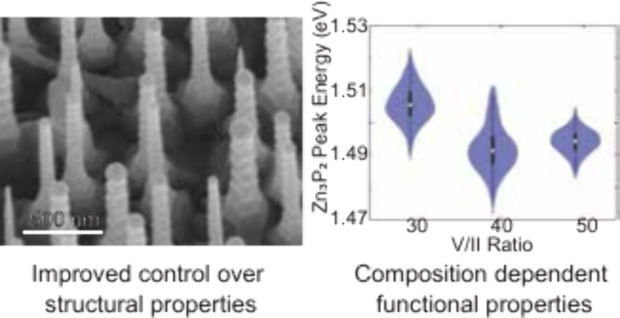

## 1 Introduction

The application of photovoltaics is an integral part in more sustainable electricity generation. The cost of installing new solar panels is continuously decreasing, and coupled with the increased demand, the roll-out of new photovoltaic modules is increasing exponentially.^1^ This development is dominated by Si-based modules, which take advantage of the maturity of the material and the established supply routes. However, the increasing demand is pushing existing polysilicon supply routes to their limit.^2^ Moreover, the indirect bandgap of Si complicates its applicability in flexible photovoltaics and other lightweight applications without the use of additional light-trapping mechanisms.^3^ Therefore, to diversify and expand the applicability of the photovoltaic sector there is currently significant research done into alternative earth-abundant semiconductors with direct band gaps, one potential candidate being zinc phosphide (Zn_3_P_2_).^4,5^

Zn_3_P_2_ has optoelectronic properties suitable for single-junction photovoltaics, such as a direct band gap of 1.5 eV,^6,7^ high optical absorptance^8^ and long carrier diffusion lengths.^9^ However, charge extraction is still challenging due to (i) its intrinsic p-doping making n-doping and p–n junctions difficult to achieve^10,11^ and (ii) lack of matching lattice parameter and coefficient of thermal expansion (CTE), which complicates Zn_3_P_2_’s incorporation in heterostructures without defect formation.^12,13^ Recently, there have been investigations that utilize various nanoscale epitaxy approaches and molecular beam epitaxy to overcome the limitations set by the lattice parameter and CTE.^14–19^ One such approach, vapor–liquid–solid (VLS) growth of epitaxial Zn_3_P_2_ nanowires, was successfully implemented to grow vertical single-crystal nanowires with tunable composition and optoelectronic properties. However, previous reports on this approach reported limited control over the growth directions and nanowire density, which is necessary to control to take advantage of their nanophotonic properties and integrate them into devices.^14,20–22^ To properly evaluate Zn_3_P_2_ nanowires potential in actual devices we therefore need to gain increased control over these parameters, and also transfer the synthesis to more large-scale and high throughput techniques, such as metalorganic chemical vapor deposition (MOCVD).

In this report we explored the necessary steps for Zn_3_P_2_ nanowire growth in MOCVD, and mapped the parameter space through a combinatorial study, focusing on temperature and partial pressures of the precursors, supported by thermodynamic modeling. Furthermore, electron microscopy is used for structural characterization and high-throughput optical spectroscopy for functional characterization to evaluate the quality and suitability of the grown material in photovoltaic applications.

## 2 Experimental Section

### 2.1 Growth

The growth was performed on InP (111)­A and B substrates (Wafer Technology Ltd.) using an Aixtron 3 × 2″ close-coupled showerhead (CCS) MOCVD system operating at a pressure of 100 mbar, constant flow rate of 8000 sccm and an InP base coverage (the effect of the base cover is expanded on in the SI). First, the native oxide was removed through degassing the samples under a PH_3_ partial pressure of 0.1 mbar for 7 min at 530 °C. Next, we deposited self-assembled In particles at 380 °C for 240 s and a TMIn partial pressure of 2.5 × 10^–3^ mbar to act as the catalyst for nanowire growth. The temperature was subsequently lowered to the set growth temperature, which ranged from 305 to 355 °C. Once the temperature had stabilized we performed a 5 min Zn predeposition step at a DEZn partial pressure of 1.0 × 10^–2^ mbar and closed PH_3_ supply. The nanowire growth was then initiated by again supplying PH_3_, with varying partial pressure depending on the experiment, while maintaining the aforementioned DEZn partial pressure. The PH_3_ partial pressures were varied from 1.25 × 10^–3^ to 0.5 mbar for growth times spanning 1–120 min.

### 2.2 Thermodynamic Calculations

The phase diagrams were calculated with the Thermo-Calc software using the same approach and data set as in ref 14, that is, by extrapolating from binary phase data from refs 23–25 .

### 2.3 Electron Microscopy

Scanning electron microscopy (SEM) was performed using a Zeiss Gemini 500 SEM operating at 5 kV using an in-lens detector. Electron transparent lamellae of longitudinal section of nanowires were obtained via focused ion beam (FIB) processing using a Helios UX 5 FIB system. A predeposition of 0.2 μm of carbon (nominal thickness) and 0.2 μm of tungsten­(W) (nominal thickness) was performed with the electron gun inside the FIB system prior to the lamella processing. This procedure aimed at limiting the contamination from the thick ion-deposited W protective layer during the final stages of the thinning. Aberration corrected HAADF-STEM images were obtained using a Thermo Fisher Scientific Spectra 300 double corrected scanning transmission electron microscope (STEM). Denoised high-magnification images were obtained by filtering the corresponding power spectra with spot masks and taking the inverse FFT. Electron Energy Loss Spectroscopy in STEM mode (EELS-STEM) compositional maps were obtained in a FEI Tecnai F20 TEM by using a GATAN QUANTUM filter. 3D atomic models were created using CaRIne Crystallography 3.1.^26^ Geometrical phase analysis (GPA) was performed on simulated and experimental images by using the licensed GPA plug-in available for Gatan Digital Micrograph.^27^

### 2.4 Photoluminescence Spectroscopy

The optoelectronic properties of the nanowires were assessed using room temperature photoluminescence (PL) spectroscopy in a confocal microscope setup, similar to that discussed in ref 28. Photoexcitation was achieved using a 532 nm continuous wave laser, with an optical power of 3 mW, focused to a diffraction limited spot with a 100× magnification objective lens. The luminescence was collected using an optical fiber and the spectrum was measured using a Horiba iHR550 spectrometer with a 150 lines/mm diffraction grating and a slit width of 1 mm. Thirty μm by 30 mm micro-PL maps were measured by translating samples in steps of 0.5 mm in *x* and *y*.

## 3 Results and Discussion

### 3.1 Nanowire Growth

Once the substrate had been degassed under a PH_3_ atmosphere in the MOCVD to remove the native oxide, the next step of the process is the deposition of the In-catalyst particles, as shown in Figure 1a. The self-assembled In particles had a density of 4.7 particles μm^–2^ with a diameter distribution of 124 ± 66 nm on InP (111)B and a density of 3.9 particles μm^–2^ with a diameter distribution of 138 ± 34 nm on InP (111)­A for identical deposition conditions. In the subsequent step the temperature was lowered to the growth temperature. In initial trials we tried to start the growth by supplying both DEZn and PH_3_ simultaneously from the start. However, this led to the results seen in the SEM image in Figure 1b, namely the formation of small nanopyramids. To promote nanowire growth (Figure 1c) we implemented a 5 min Zn predeposition step. To understand the importance of this step we calculated the isothermal section of the Zn–In–P ternary phase diagram for our standard growth temperature (330 °C) shown in Figure 1d. To understand the initial nanowire growth mechanism we closely observe the corner closest to pure In (bottom left, zoomed plot in Figure 1e). We observe that if we were to provide Zn and P to the system we will enter a phase region where the stable phases are L + InP, meaning that we would start consuming our catalyst particle to precipitate InP, which explains the tapered shaped nanopyramids observed in Figure 1b. On the other hand, by first providing Zn through the predeposition we instead move into the top phase region in Figure 1e, which consists of L + Zn_3_P_2_, where Zn_3_P_2_ nanowire growth can be achieved, which is consistent with the SEM data shown in Figure 1c.

**Figure 1:**
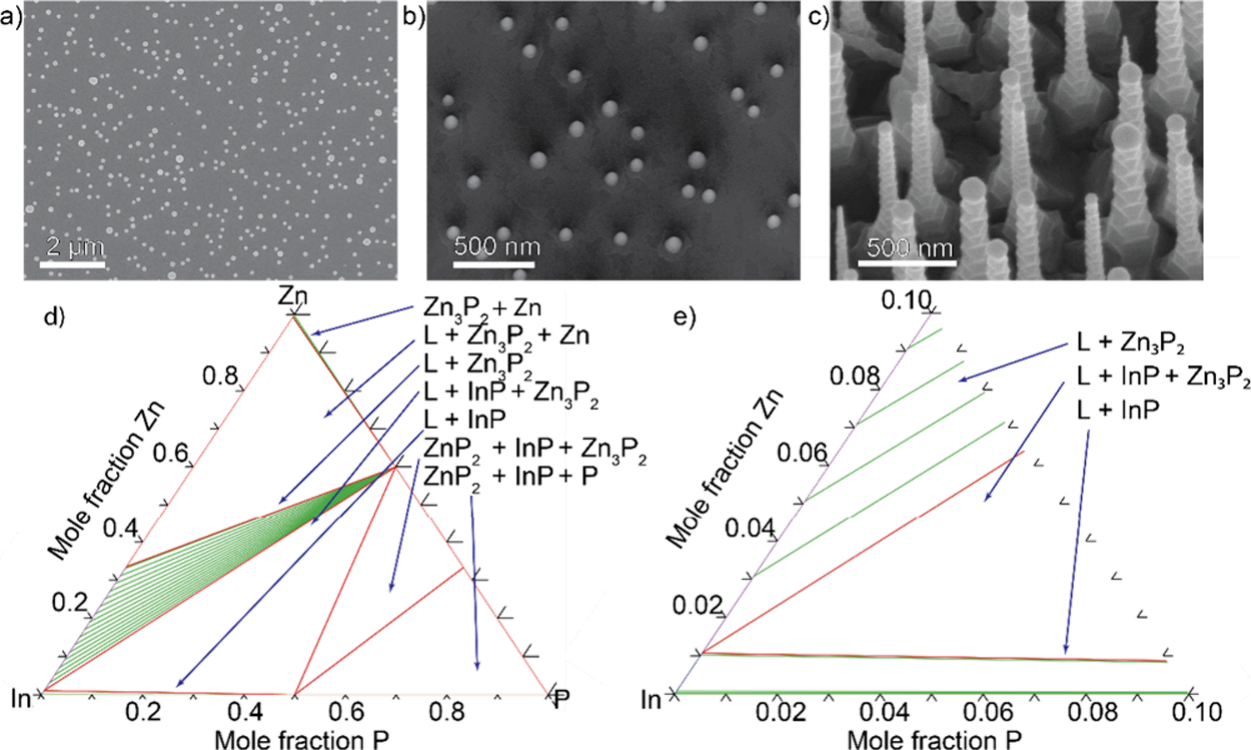
Scanning electron microscopy images of (a) as-deposited In nanoparticles on InP (111)B (top view), (b) pyramids grown without Zn predeposition (15° tilt), and (c) nanowires grown with Zn predeposition (30° tilt). (d) Shows the calculated Zn–In–P ternary phase diagram at 330 °C and (e) shows a zoomed in section closest to pure In.

Another aspect worth noting is that we do not observe the coexistence of ZnP_2_ and a liquid in the ternary phase diagram. This would explain why in In-catalyzed Zn_3_P_2_ nanowires there have been no observation of the other stoichiometry irrespective of growth conditions in this or previous studies (although for different catalyst materials, such as Bi, it has been demonstrated).^14,29–31^

We explored the growth parameter space by tuning the V/II ratio and temperature at constant DEZn partial pressure (1.0 × 10^–2^ mbar) and temperature (305–355 °C). First, we varied the PH_3_ partial pressure from 1.25 × 10^–3^ to 0.5 mbar, corresponding to nominal V/II ratios of 1.25–50.1 at a growth temperature of 336 °C. What we observed at lower PH_3_ partial pressures was a low density of short nanowires with no obvious tapering of the diameter, as shown in Figure 2a. However, with increasing PH_3_ we initially saw a significant increase of the growth rate, up to a PH_3_ partial pressure of 0.25 mbar (V/II ratio of 25.1). For even higher PH_3_ flows we still saw significant axial nanowire growth, but the tapering due to radial vapor–solid (VS) growth started to increase. We also started to observe the absence of the catalyst particle on some of the nanowires (see Figure 2a at V/II of 45.1 and Figure S1). This could possibly have occurred through consumption of the catalyst particle due to the high PH_3_ partial pressure, and results in a stop in the axial growth partway through the run as shown in Figure S1.

**Figure 2:**
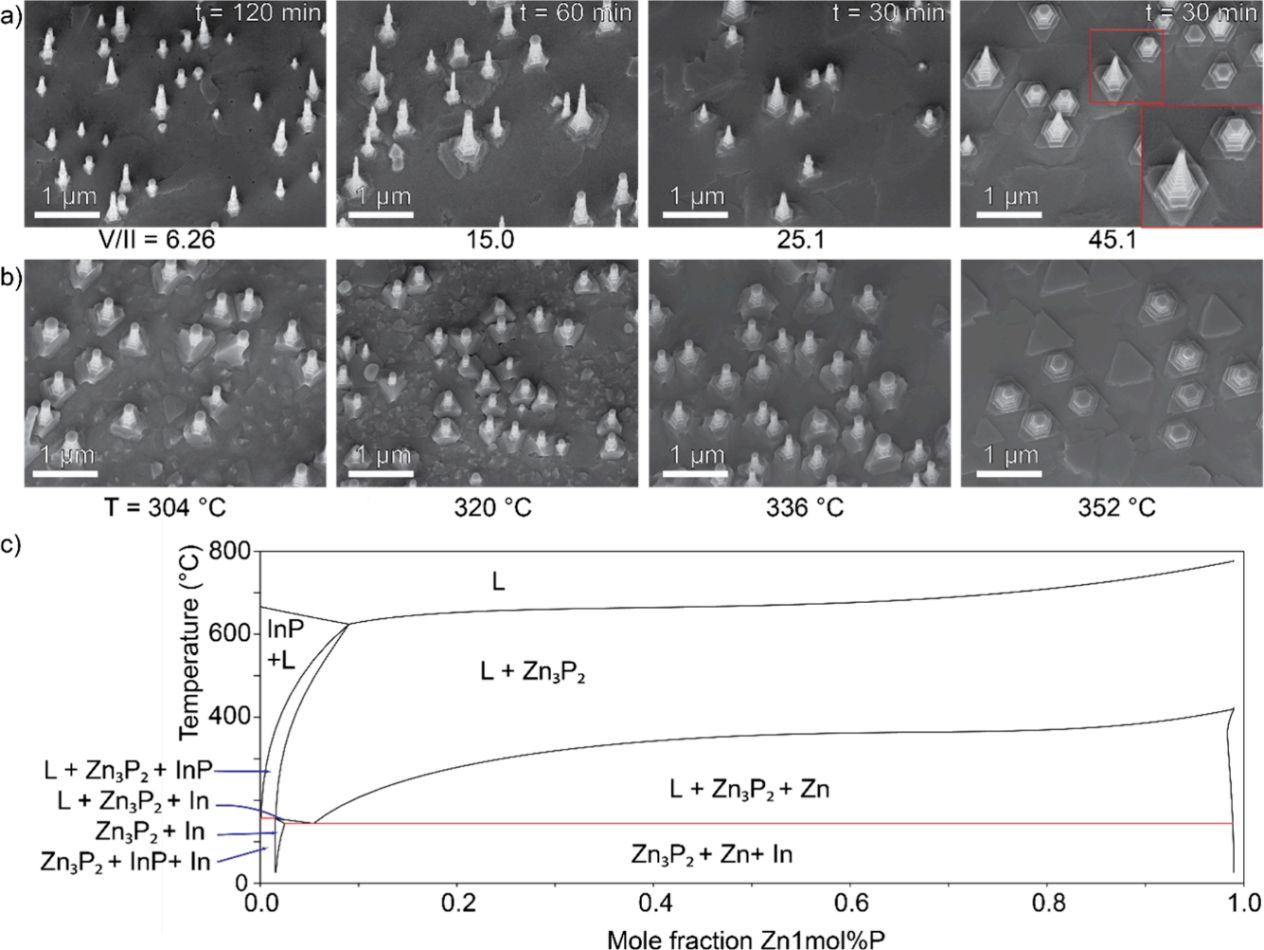
(a) 15° tilted SEM images of the V/II series at 336 °C and constant Zn flow, with an inset in V/II ratio of 45.1 image highlighting (left) a wire with a catalyst particle and (right) a nanowire without a particle. (b) 15° tilted SEM images of temperature series at a constant V/II ratio of 35.1. (c) Plot of the calculated pseudobinary phase diagrams of the Zn1 mol %P–In1 mol %P system.

When we instead explored the effect of temperature for a constant V/II ratio (PH_3_ partial pressure of 0.35 mbar, V/II ratio of 35.1), we saw that increasing the temperature rapidly suppressed the growth of nanowires. As can be seen in Figure 2b, we observe the formation of small stub-like structures and we could not observe any catalyst particles. This is attributed to the increased desorption of the Zn atoms on the surface in combination with the increased cracking efficiency of PH_3_, leading to a much higher local V/II ratio at elevated temperatures. Consequently, the drastic change in local conditions will reduce the diffusion length of Zn and increase the probability for nucleation away from the catalyst particle, which will limit the VLS growth in favor of VS growth. This results in preferential radial overgrowth and thin film formation between nanowires as opposed to axial VLS growth. The increased temperature and decreased Zn also has the potential to push the growth into phase regions which allow for the formation of InP (to the right corner of the phase diagrams shown in Figure 1d,e), which would consume the droplet and again limit the window for sustained VLS growth. On the other hand, by lowering the temperature we continued to observe nanowire growth in the explored range. However, the growth rate was decreasing significantly with decreasing temperature. When going from a growth temperature of 336–304 °C we observed a decrease of average nanowire length from 1050 ± 62 to 800 ± 83 nm for a growth time of 30 min. This is most likely related to the decrease in the PH_3_ cracking efficiency in this temperature range, which affects the material supply and growth rate.^32^

The temperatures and V/II ratios needed for MOCVD growth of Zn_3_P_2_ were higher than those reported in previous literature for growth using molecular beam epitaxy (MBE). We expect that the difference in growth temperature between the techniques is related to the decomposition of the PH_3_, setting the lower limit of the parameter window for MOCVD. However, while there is decomposition of the precursors even at the lower end of the explored temperature range, the low efficiency results in the need for high PH_3_ partial pressures to compensate for the lowered decomposition efficiency. This may contribute to the need for the higher nominal V/II ratios in MOCVD compared to MBE in the initial material supply. However, this will not necessarily result in a large difference in the local V/II ratio at the growth interface due to the low utilization of the PH_3_. To better understand the growth window, we can extract the pseudobinary Zn–In phase diagram with a constant P concentration of 1% to simulate the composition of the droplet during growth, presented in Figure 2c. In this phase diagram we can observe a growth window which contains a liquid phase and a Zn_3_P_2_ solid phase (L + Zn_3_P_2(s)_ region). For Zn concentrations in the order of a few percent in the droplet, the growth temperature can in theory go as low as 140 °C while the upper limit is set by the transition into the L + InP_(s)_ + Zn_3_P_2(s)_ region where the In making up the droplet would be consumed to form InP. As seen in the temperature series in Figure 2b, we start crossing into this region before 350 °C, we can estimate that we have approximately ∼3 to 5% Zn in the droplet during growth. However, as nanowire growth occurs out of equilibrium (where the phase diagram is calculated) due to the constant supply of reactants, there is a significant uncertainty in this estimate. For higher Zn concentrations the growth window moves up in temperature to avoid the formation of Zn (lower limit) and because it is less favorable to form InP (upper limit). This does indicate that the theoretical growth window for Zn_3_P_2_ nanowires is very extensive, however, practical limitations with precursor supplies, mainly Zn re-evaporation and PH_3_ cracking, limit the experimental parameter space by MOCVD.

Another big difference between the current and previous studies is that we could significantly suppress the appearance of multiple morphologies of epitaxial Zn_3_P_2_ nanowires in the same experiment as observed previously.^14^ In previous reports, the catalyst particles were generated through the decomposition of the InP substrate.^14^ However, in this study we developed a separate deposition step of the catalyst particle, which maintains a pristine interface that in turn favors only one type of growth. As schematically illustrated in Figure 3a, previous approaches could potentially expose multiple possible facets that could initiate nanowire growth, resulting in a mixed morphology distribution, similar to what was observed for InP nanowires growing on InP (001).^33^ On the InP (111)­A/B substrates used in this study we mainly observed the zigzag heterotwin-plane superlattice nanowires growing perpendicular to the substrate and the (101) plane of the Zn_3_P_2_.^34^ In theory, we should have been able to access a twin-free morphology as described in ref 14 by lowering the temperature. However, due to the lower temperature limit set by the PH_3_ decomposition we were unable to observe them experimentally. Only at very low V/II ratios, such as those shown in Figure 2a, we could observe short segments without the zigzag morphology. We did however observe a new morphology of nanowires, shown in Figure 3b,c. These grew at an angle of 76° with respect to the surface of the InP (111)­A/B substrates. We believe this nanowire morphology is the result of a defect formation in the initial nucleus explained in more detail below.

**Figure 3:**
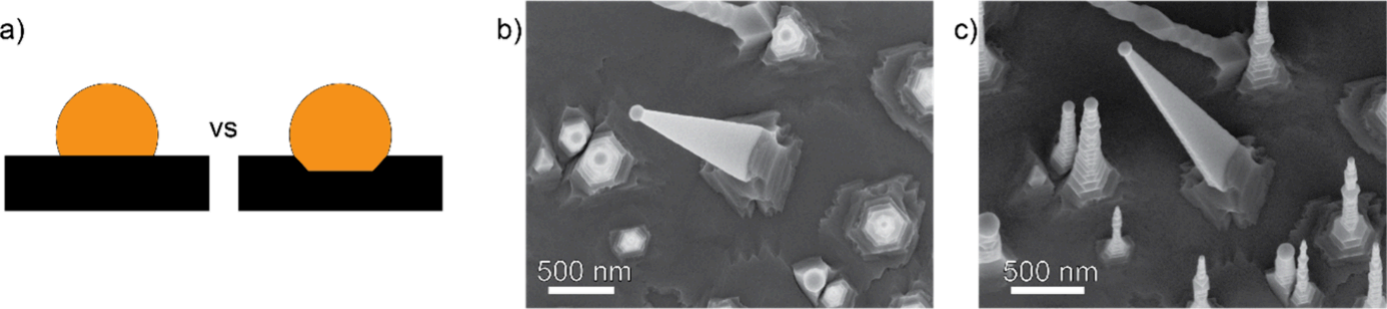
(a) Diagram depicting nucleation on flat vs mixed facet. SEM images of the new morphology showing (b) top view and (c) 15° tilted view.

### 3.2 Structural Characterization

We investigated the epitaxial relation between the Zn_3_P_2_ nanowires and the InP (111) A and B surfaces, as well as the defect structure using AC-HAADF-STEM. Images of the interfaces with InP (111) of different polarity and the base of a nanowire are presented in Figure 4a,b. We have observed that the Zn_3_P_2_ adapts with a good lattice match and clear epitaxial relation to the InP below. However, we have observed small differences depending on the polarity selected. In A-polar InP the surface is terminated with In, while in B-polar it is terminated with P. In the A-polar case, it seems there is an In–Zn interface, while in the case of the B-polar, the interface looks more like P–Zn (truncated unit cell). Moreover, we consistently observed a twin plane close to the interface between the InP (111)­A and Zn_3_P_2_ nanowires, as highlighted in the higher magnification atomic resolution image in Figure 4c. However, this is not observed for growth on InP (111)­B (Figure 4d) or in the thin film that grew in the regions between nanowires. In VLS growth of III–V and II–VI materials, one of the polarities has an increased probability to show twinning, especially in conditions out of steady-state (initial and final stages of nanowire growth).^35^ In addition, the formation of twins predominantly happens in one of the polarities.^35^ In GaAs it has been demonstrated that twinning predominantly occurs for nanowires growing along ⟨111⟩ B, but rarely when grown along ⟨111⟩ A.^36,37^ In other systems, like GaSb^38^ or InP,^39,40^ the opposite trend have been reported, with twins more readily forming in nanowires growing along ⟨111⟩ A (without the presence of dopants).^39^ In the present case, we suspect that initial nucleation from the In-rich catalyst particle, when the growth process starts, may first produce a twinned InP layer before the Zn_3_P_2_ growth. In A-polar substrates, this first InP layer precipitates out of steady-state (initial growth stages), so, with high probability of twinning (as observed). In the B-polar case, this phenomenon would be rare as shown in the present experimental results.

**Figure 4:**
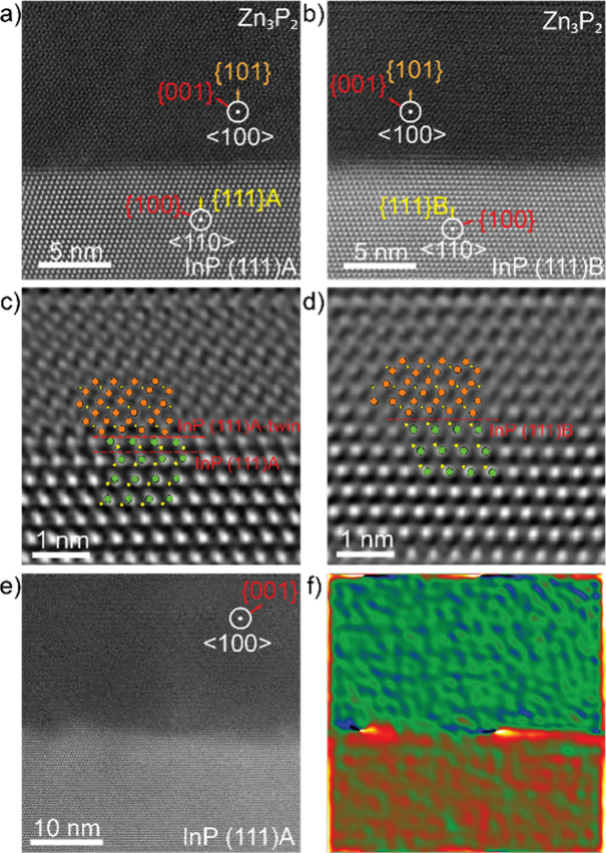
AC HAADF-STEM images of the interface between (a) InP (111)­A or (b) InP (111)B and Zn_3_P_2_ viewed along a ⟨110⟩ zone axis with respect to the InP (⟨100⟩ with respect to the Zn_3_P_2_). Filtered and high-magnification images of the interfaces of (c) InP (111)­A and (d) InP (111)B and Zn_3_P_2_ nanowires highlighting the twin and interface atomic structure. (e) Atomic resolution AC HAADF STEM image of the InP (111)­A substrate and thin film between nanowires and corresponding GPA map (f) highlighting the misfit dislocations.

To investigate the residual strain in the nanowire we performed geometric phase analysis (GPA) and 3D modeling on Zn_3_P_2_ grown on InP (111)­B, compiled in Table 1. From the GPA we could not observe any misfit dislocations or other regular interface-related defects (Figure S2). However, we observe that the Zn_3_P_2_ close to the interface experiences slight strain. When the nanowires grow the Zn_3_P_2_ becomes progressively more relaxed, as explained by the radial strain relaxation observed in nanowires. The relaxation is observed for both nanowire morphologies. From the GPA analysis we also estimate the relative distortions in terms of dilation and rotation of the nanowires with respect to the substrate, as well as the magnitude of plane mismatch by considering the parallel and perpendicular planes with respect to the InP (111) surface, namely the (011) and (100) planes of Zn_3_P_2_. From this we can also calculate the strain tensor components ε_*xx*_ and ε_*yy*_, corresponding to the parallel and perpendicular directions with respect to the substrate, respectively. The residual values of the plane mismatch computed considering the (011) Zn_3_P_2_ plane indicate a minimal compression within the Zn_3_P_2_ layer. Along the (100) Zn_3_P_2_ plane, perpendicular to the interface, we instead obtained a positive value of the residual plane mismatch. The positive value indicates an overall expansion of the Zn_3_P_2_ layer in this direction. The rotation of Zn_3_P_2_ relative to the substrate is negligible for both the parallel and perpendicular directions.

**Table 1:** Summary of GPA Results along Different Directions and the Residual Strain of Zn_3_P_2_ Nanowires Grown on InP (111)­B

	plane mismatch			
direction	(011)_Zn_3_P_2__//(111)_I__nP_ (%)	(100)_Zn_3_P_2__//(1–10)	ε	ε
GPA measured	–3.0	–2.3	–2.3	–2.9
fully relaxed (theory)	–2.9	–2.7	–2.6	–2.9
residual strain	–0.1	0.4	0.3	0.0

We also performed the same analysis on the Zn_3_P_2_ grown on InP (111)­A. However, the distorted interface under the nanowires due to the twins did not yield any useful quantitative results. Instead, the comparison with the area growing between nanowires turned out to be more interesting, shown in Figure 4e,f. In the absence of the surface rearrangement observed at the base of the nanowires, we could instead observe the formation of defects at the interface between the thin film and the substrate as shown by the GPA (Figure 4f), which are discussed in more detail in the SI (see Figure S3 and corresponding discussion). This would indicate that the rearrangement of the interface under the nanowire and the stress relaxation are useful mechanisms in mitigating defect formation even at significantly greater diameters compared to those proposed previously.^17,41^

We extended the HAADF-STEM analysis to the bulk of the tilted nanowires discussed at the end of Section 3.1, shown in Figure 5. These nanowires grew with a tilt with respect to the InP (111) surface and without the twin plane superlattice structure. However, we observed multiple types of defects within these nanowires. First, we noted a twin with a similar structure to previously observed heterotwins, running along the growth axis of the nanowire in a not previously observed configuration, shown in Figure 5a–c.^34^ We observed it reaching very close, but not all the way to the InP-Zn_3_P_2_ interface in our FIB cross sections. However, due to the nature of the fabrication process we removed a significant part of the nanowire in the milling step to achieve the electron transparent sample, and there is a possibility for the defect to reach all the way down to the interface in these areas. We also noticed an increased presence of this morphology along surface defects, as shown in Figure S4, however, we could not tie the formation of the tilted morphology exclusively to surface defects. Moreover, we also observed multiple rotated domains, similar to those observed in ref 16, forming all along the nanowire.

**Figure 5:**
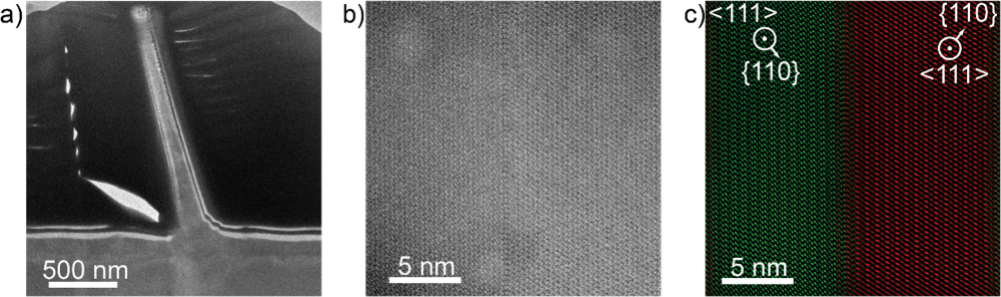
(a) Low-magnification view of a cross-section of the new morphology of nanowire. (b) AC HAADF-STEM of the heterotwin running along the axis of the nanowire in (a). (c) Color-coded image based on the filtered FFT image of (b), highlighting the different rotations on the different sides of the twin.

### 3.3 Optoelectronic Characterization

To evaluate the optoelectronic properties of the Zn_3_P_2_ nanowires we performed PL mapping of 3 samples grown at 336 °C at different V/II ratios on InP (111)B substrates as the higher nanowire density was necessary to observe sufficient PL signal. An example PL spectrum from Zn_3_P_2_ nanowires grown with a V/II ratio of 46.3 is shown in Figure 6a. The spectrum contains two features: the dominant peak at 1.35 eV is consistent with band-edge recombination in InP and is therefore attributed to carrier recombination in the InP substrate.^42^ The high energy side of the InP peak can be described by an exponential decay via a Boltzmann distribution.^43^ Therefore, to isolate the emission from the nanowires, the spectrum was fit with an empirical model, described by eq 1, for energies larger than 1.38 eV. On the high energy side of this peak there is a broad shoulder which is 2 orders of magnitude lower in intensity. This shoulder has a peak energy of around 1.5 eV which is comparable to previous studies of Zn_3_P_2_ at room temperature–we therefore attribute this peak to recombination from the Zn_3_P_2_ nanowires.^7,9,15^ The comparatively low emission intensity is partially attributed to the reduced volume of Zn_3_P_2_ when compared with the substrate, with an additional contribution from inefficient carrier recombination in the nanowires, potentially due to nonradiative recombination at surface states and planar or point defects related to off-stoichiometric composition.^7,14^

**Figure 6:**
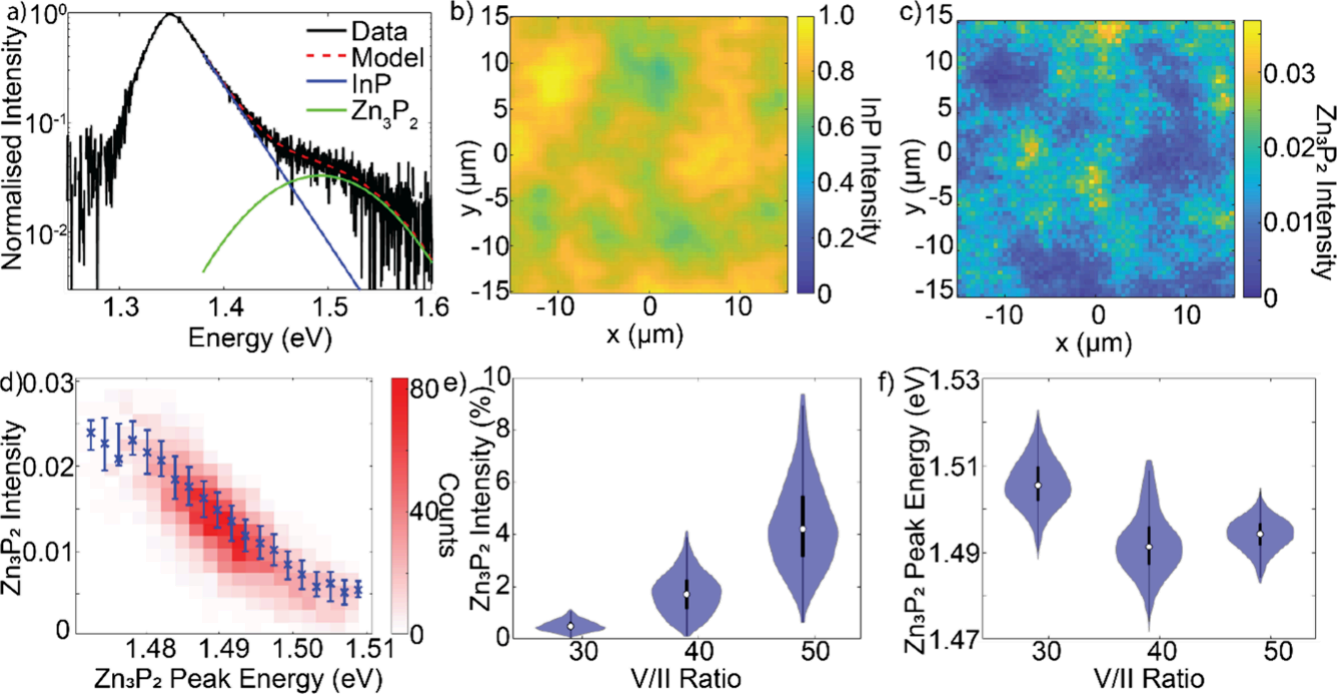
Room temperature photoluminescence of Zn_3_P_2_ nanowires on InP substrates. (a) Normalized emission spectrum with emission peaks from the substrate and the nanowires, eq 1 has been fit to the data for energies greater than 1.38 eV. (b) Emission intensity map of the InP substrate. (c) Emission intensity of the Zn_3_P_2_ nanowire peak, normalized to the maximum InP emission. (d) 2D histogram showing the correlation between the nanowire peak energy and emission intensity. The blue markers represent the median values in each horizontal bin, with the error bars indicating the interquartile range. The Pearson’s linear regression coefficients for this correlation are *r* = −0.81, *p* ∼ 0. (e, f) Violin plots showing the distribution of the nanowire emission intensity and peak energy for samples grown with different V/II ratios.

To investigate the spatial dependence of this peak, the spectrum was fit was fit with an empirical model, described by eq 1:

$$I_{\mathrm{PL}}(E)=A_{\mathrm{InP}}\exp(-k_{\mathrm{InP}}(E-E_{\mathrm{InP}}))+A_{{\mathrm{Zn}}_{3}P_{2}}\exp\left(-\frac{(E-E_{{\mathrm{Zn}}_{3}P_{2}}{)}^{2}}{\sigma^{2}}\right)$$ 1

where the tail of the InP is represented by an exponential decay, which has an amplitude of *A*_InP_ at an energy of *E*_InP_ and a decay constant *k*_InP_. The higher energy emission is described by a Gaussian with amplitude *A*_Zn_3_P_2__, centered at energy *E*_Zn _3_P_2__ and with width σ. The spatial map of the normalized integrated intensity of the two peaks are shown in Figure 6a,b, respectively. The InP intensity varies by up to 50% over the studied area with correlated regions of 5–10 μm in size. In contrast, the Zn_3_P_2_ intensity changes from zero to 3.5% of the total intensity and is negatively correlated with the InP intensity. This pattern is consistent with a variable nanowire density across the InP substrate: regions of higher nanowire density result in greater photon absorption in the nanowires and thus a higher nanowire emission intensity, along with less absorption in the underlying InP and thus a reduction in the InP emission intensity.

The emission intensity is associated with other optoelectronic properties of the nanowires. Figure 6d shows a strong negative correlation between the emission intensity and the peak photon energy. The peak energy blueshifts by 40 meV across the data set, and this corresponds to a drop in emission intensity by close to 100%. In Zn_3_P_2_, peak energy shifts have been previously linked to changes in the crystal composition, and these results suggest that this composition is also connected to the carrier recombination efficiency.^7^

To investigate this effect further, an additional two samples were grown with differing V/II ratios. Micro-PL maps were measured from each of these samples and are qualitatively similar in their spatial dependence to those in Figure 6b,c. Despite this, there are stark differences in intensity and emission energy for each of these samples. Figure 6e shows a strong positive relationship between the median Zn_3_P_2_ intensity and the V/II ratio, which increases from 0.5 to 4.2% across the range. The interquartile range is approximately 60% of the median intensity for each sample, implying that while the median intensity increases, the shape of the distribution remains consistent for all three samples. SEM image analysis of these samples demonstrate that there is no significant difference in the nanowire density–it is therefore likely that this intensity difference is related to the radiative recombination efficiency. This result implies that, as the V/II ratio is increased during growth, the resultant nanowires have fewer defects and thus the nonradiative recombination rate is reduced in the investigated range. To determine the exact defect transition suppressed by PL spectroscopy one would have to investigate the low temperature PL in the energy range where the InP substrate luminesces, which therefore was not feasible for the Zn_3_P_2_ nanowire samples.^7,9^

Figure 6f shows that the V/II ratio also has an impact on the emission energy of the nanowires: the median energy changes from 1.505, to 1.491 and to 1.494 eV, with increasing V/II ratio. While this is a nonmonotonic behavior, the 13 meV shift is statistically significant and suggests a change in the crystal composition and functional properties with V/II ratio. Furthermore, the interquartile range reduces from 8 to 5 meV for the largest V/II ratio studied–suggesting that the crystal uniformity based on the radiative recombination efficiency of the nanowires is improved under these conditions.

## 4 Conclusions

In this study we have demonstrated the steps to grow Zn_3_P_2_ nanowires with high morphological selectivity using In as a catalyst. Furthermore, we have demonstrated improved morphological control over the Zn_3_P_2_ nanowires by introducing a separate particle deposition step. We found it imperative to load the particles with Zn before providing P to avoid consumption of the catalyst and formation of unwanted phases. Using this approach we have mapped out the parameter space to achieve epitaxial growth of Zn_3_P_2_ nanowires on InP (111)­A/B substrates using MOCVD and relating it to calculated phase diagrams and experimental limitations, such as PH_3_ cracking (low T) and Zn re-evaporation (high T).

We performed structural analysis by investigating FIB cross sections with AC HAADF-STEM, showing that Zn_3_P_2_ nanowires readily grow on either A or B polar InP (111) substrates. However, twins consistently form in the top layers of the InP (111)­A substrates at the base of the nanowires. We observed misfit dislocations at the interface of the thin films growing between nanowires and the substrate, while none were observed at the interface under the nanowires. Moreover, to evaluate the strain due to lattice mismatch we performed GPA. The analysis showed that the nanowire had been able to almost completely accommodate the strain due to lattice mismatch, exhibiting a lattice parameter close to the relaxed bulk lattice parameter from literature. We also investigated a new morphology of nanowires, growing with a rectangular cross section and at an angle to the InP (111) A/B substrate surfaces. These nanowires exhibited a high concentration of defects, such as rotated domains, and heterotwin along the longitudinal axis of the nanowire. The origin of this morphology is suspected to be linked to surface defects in the substrate.

Through PL spectroscopy we investigated the effect of V/II ratio on the functional properties of the Zn_3_P_2_ nanowires. First, for all samples we observed PL from the Zn_3_P_2_ nanowires in the region of 1.5 eV, which is in the range optimal for single-junction solar cells. For the explored V/II range we also observed an increase in PL intensity for samples grown at higher V/II ratio, suggesting that the carrier recombination efficiency in Zn_3_P_2_ is related to the stoichiometry. Furthermore, we observed a decrease of the room temperature bandgap recombination by 13 meV when going from a V/II ratio of 28–38, further highlighting the importance of composition in determining the functional properties of Zn_3_P_2_ nanowires.

## Supplementary Material



## Notes

Comments regarding influence of chamber history on growth; SEM images showing the lack of catalyst particles at high V/II ratios and the increased presence of tilted nanowires along surface defects; and additional HAADF STEM images GPA maps of the Zn_3_P_2_/InP interface (PDF)

## References

[1] Victoria M., Haegel N., Peters I. M., Sinton R., Jäger-Waldau A., del Cañizo C., Breyer C., Stocks M., Blakers A., Kaizuka I., Komoto K., Smets A. (2021). Solar Photovoltaics
Is Ready to Power a Sustainable Future. Joule.

[2] Bahar, H. ; Abdelilah, Y. ; Briens, F. ; Bojek, P. ; Criswell, T. ; Kurumi, K. ; Moorhouse, J. ; Rodriguez, G. ; Veerakumar, K. Special Report on Solar PV Global Supply Chains; International Energy Agency, 2022.

[3] Tavakoli N., Spalding R., Lambertz A., Koppejan P., Gkantzounis G., Wan C., Röhrich R., Kontoleta E., Koenderink A. F., Sapienza R., Florescu M., Alarcon-Llado E. (2022). Over 65% Sunlight
Absorption in a 1 Μm Si Slab with Hyperuniform Texture. ACS Photonics.

[4] Escobar
Steinvall S., Stutz E. Z., Paul R., Zamani M., Leran J.-B., Dimitrievska M., Fontcuberta i Morral A. (2022). Nanoscale
Growth Initiation as a Pathway to Improve the Earth-Abundant Absorber
Zinc Phosphide. ACS Appl. Energy Mater..

[5] Lewis N. S. (2016). Research
Opportunities to Advance Solar Energy Utilization. Science.

[6] Dimitrievska M., Hage F. S., Escobar
Steinvall S., Litvinchuk A. P., Stutz E. Z., Ramasse Q. M., Fontcuberta i Morral A. (2021). The Advantage
of Nanowire Configuration in Band Structure Determination. Adv. Funct. Mater..

[7] Stutz E. Z., Ramanandan S. P., Paul R., Flor M., Zamani M., Escobar Steinvall S., Sandoval Salaiza D. A., Xifra C., Spadaro M. C., Leran J.-B., Litvinchuk A. P., Arbiol J., Fontcuberta
i Morral A., Dimitrievska M. (2022). Stoichiometry Modulates the Optoelectronic
Functionality in Zinc Phosphide (Zn3-XP2+x). Faraday Discuss..

[8] Swinkels M. Y., Campo A., Vakulov D., Kim W., Gagliano L., Escobar Steinvall S., Detz H., De Luca M., Lugstein A., Bakkers E., Fontcuberta i Morral A., Zardo I. (2020). Measuring
the Optical Absorption of Single Nanowires. Phys. Rev. Appl..

[9] Kimball G. M., Mueller A. M., Lewis N. S., Atwater H. A. (2009). Photoluminescence-Based
Measurements of the Energy Gap and Diffusion Length of Zn3P2. Appl. Phys. Lett..

[10] Catalano A., Hall R. (1980). Defect Dominated Conductivity in Zn3P2. J.
Phys. Chem. Solids.

[11] Yuan Z., Xiong Y., Hautier G. (2023). First-Principles Study
of Intrinsic
and Hydrogen Point Defects in the Earth-Abundant Photovoltaic Absorber
Zn3P2. J. Mater. Chem. A.

[12] Long J. (1983). The Growth
of Zn3P2 by Metalorganic Chemical Vapor Deposition. J. Electrochem..

[13] Zamani M., Stutz E. Z., Escobar
Steinvall S., Zamani R. R., Paul R., Leran J.-B., Dimitrievska M., Fontcuberta i Morral A. (2021). The Path towards
1 Μm Monocrystalline Zn3P2 Films on InP: Substrate Preparation,
Growth Conditions and Luminescence Properties. J. Phys.: Energy.

[14] Escobar
Steinvall S., Tappy N., Ghasemi M., Zamani R. R., LaGrange T., Stutz E. Z., Leran J.-B., Zamani M., Paul R., Fontcuberta i Morral A. (2020). Multiple Morphologies
and Functionality of Nanowires Made from Earth-Abundant Zinc Phosphide. Nanoscale Horiz..

[15] Escobar
Steinvall S., Stutz E. Z., Paul R., Zamani M., Dzade N. Y., Piazza V., Friedl M., de Mestral V., Leran J.-B., Zamani R. R., Fontcuberta i Morral A. (2021). Towards Defect-Free
Thin Films of the Earth-Abundant Absorber Zinc Phosphide by Nanopatterning. Nanoscale Adv..

[16] Spadaro M. C., Escobar Steinvall S., Dzade N. Y., Martí-Sánchez S., Torres-Vila P., Stutz E. Z., Zamani M., Paul R., Leran J.-B., Fontcuberta i Morral A., Arbiol J. (2021). Rotated Domains
in Selective Area Epitaxy Grown Zn3P2: Formation Mechanism and Functionality. Nanoscale.

[17] Glas F. (2006). Critical Dimensions
for the Plastic Relaxation of Strained Axial Heterostructures in Free-Standing
Nanowires. Phys. Rev. B.

[18] Chi C.-Y., Chang C.-C., Hu S., Yeh T.-W., Cronin S. B., Dapkus P. D. (2013). Twin-Free GaAs Nanosheets
by Selective Area Growth:
Implications for Defect-Free Nanostructures. Nano Lett..

[19] Bosco J. P., Kimball G. M., Lewis N. S., Atwater H. A. (2013). Pseudomorphic Growth
and Strain Relaxation of Alpha-Zn3P2 on GaAs(001) by Molecular Beam
Epitaxy. J. Cryst. Growth.

[20] Krogstrup P., Jo̷rgensen H. I., Heiss M., Demichel O., Holm J. V., Aagesen M., Nygard J., Fontcuberta i Morral A. (2013). Single-Nanowire
Solar Cells beyond the Shockley–Queisser Limit. Nat. Photonics.

[21] Heiss M., Russo-Averchi E., Dalmau-Mallorquí A., Tütüncüoğlu G., Matteini F., Rüffer D., Conesa-Boj S., Demichel O., Alarcon-Lladó E., Morral A. F. i. (2013). III–V
Nanowire Arrays: Growth and Light Interaction. Nanotechnology.

[22] Mann S. A., Grote R. R., Osgood R. M., Alu A., Garneet E. C. (2016). Opportunities and Limitations for
Nanophotonic Structures
To Exceed the Shockley-Queisser Limit. ACS Nano.

[23] Lee B.-J. (1996). Thermodynamic
Assessments of the Sn-Zn and In-Zn Binary Systems. Calphad.

[24] Ansara I., Chatillon C., Lukas H. L., Nishizawa T., Ohtani H., Ishida K., Hillert M., Sundman B., Argent B. B., Watson A., Chart T. G., Anderson T. (1994). A Binary Database
for III–V Compound Semiconductor Systems. Calphad.

[25] Ghasemi M., Stutz E., Escobar Steinvall S., Zamani M., Fontcuberta
i Morral A. (2019). Thermodynamic Re-Assessment of the Zn-P Binary System. Materialia.

[26] Boudias, C. ; Monceau, D. CaRIne Crystallography: The Crystallographic Software for Research and Teaching; CaRIne Crystallography, 2006.

[27] Hÿtch M. J., Snoeck E., Kilaas R. (1998). Quantitative Measurement
of Displacement
and Strain Fields from HREM Micrographs. Ultramicroscopy.

[28] Church S. A., Patel N., Al-Abri R., Al-Amairi N., Zhang Y., Liu H., Parkinson P. (2023). Holistic Nanowire
Laser Characterization as a Route to Optimal Design. Advanced Optical Materials.

[29] Oh S. H., Kim Y. (2021). Cubic ZnP2 Nanowire
Growth Catalysed by Bismuth. CrystEngComm.

[30] Kim H. S., Myung Y., Cho Y. J., Jang D. M., Jung C. S., Park J., Ahn J.-P. (2010). Three-Dimensional
Structure of Twinned
and Zigzagged One-Dimensional Nanostructures Using Electron Tomography. Nano Lett..

[31] Im H. S., Park K., Jang D. M., Jung C. S., Park J., Yoo S. J., Kim J.-G. (2015). Zn3P2-Zn3As2
Solid Solution Nanowires. Nano Lett..

[32] Stringfellow, G. B. Organometallic Vapor-Phase Epitaxy: Theory and Practice; Elsevier, 1999.

[33] Krishnamachari U., Borgstrom M., Ohlsson B. J., Panev N., Samuelson L., Seifert W., Larsson M. W., Wallenberg L. R. (2004). Defect-Free
InP Nanowires Grown in [001] Direction on InP (001). Appl. Phys. Lett..

[34] Escobar
Steinvall S., Ghisalberti L., Zamani R. R., Tappy N., Hage F. S., Stutz E. Z., Zamani M., Paul R., Leran J.-B., Ramasse Q. M., Carter W. C., Fontcuberta
i Morral A. (2020). Heterotwin Zn3P2 Superlattice Nanowires: The Role of
Indium Insertion in the Superlattice Formation Mechanism and Their
Optical Properties. Nanoscale.

[35] de
la Mata M., Zamani R. R., Martí-Sánchez S., Eickhoff M., Xiong Q., Fontcuberta i Morral A., Caroff P., Arbiol J. (2019). The Role of Polarity in Nonplanar
Semiconductor Nanostructures. Nano Lett..

[36] Zamani M., Imbalzano G., Tappy N., Alexander D. T. L., Martí-Sánchez S., Ghisalberti L., Ramasse Q. M., Friedl M., Tütüncüoglu G., Francaviglia L., Bienvenue S., Hébert C., Arbiol J., Ceriotti M., Fontcuberta i Morral A. (2020). 3D Ordering
at the Liquid–Solid Polar Interface of Nanowires. Adv. Mater..

[37] Zamani M., Tütüncüoglu G., Martí-Sánchez S., Francaviglia L., Güniat L., Ghisalberti L., Potts H., Friedl M., Markov E., Kim W., Leran J.-B., Dubrovskii V. G., Arbiol J., Fontcuberta
i Morral A. (2018). Optimizing the Yield of A-Polar GaAs Nanowires to Achieve
Defect-Free Zinc Blende Structure and Enhanced Optical Functionality. Nanoscale.

[38] Zamani R. R., Gorji Ghalamestani S., Niu J., Sköld N., Dick K. A. (2017). Polarity and Growth Directions in
Sn-Seeded GaSb Nanowires. Nanoscale.

[39] Ikejiri K., Ishizaka F., Tomioka K., Fukui T. (2012). Bidirectional Growth
of Indium Phosphide Nanowires. Nano Lett..

[40] Ikejiri K., Kitauchi Y., Tomioka K., Motohisa J., Fukui T. (2011). Zinc Blende
and Wurtzite Crystal Phase Mixing and Transition in Indium Phosphide
Nanowires. Nano Lett..

[41] Cirlin G. E., Dubrovskii V. G., Soshnikov I. P., Sibirev N. V., Samsonenko Yu. B., Bouravleuv A. D., Harmand J. C., Glas F. (2009). Critical Diameters
and Temperature Domains for MBE Growth of III-V Nanowires on Lattice
Mismatched Substrates. Phys. Status Solidi RRL.

[42] Bugajski M., Lewandowski W. (1985). Concentration-dependent Absorption and Photoluminescence
of N-type InP. J. Appl. Phys..

[43] Davies C. L., Parkinson P., Jiang N., Boland J. L., Conesa-Boj S., Tan H. H., Jagadish C., Herz L. M., Johnston M. B. (2015). Low Ensemble
Disorder in Quantum Well Tube Nanowires. Nanoscale.

[44] Ciancio R., Dunin-Borkowski R. E., Snoeck E., Kociak M., Holmestad R., Verbeeck J., Kirkland A. I., Kothleitner G., Arbiol J. (2022). E-DREAM: The European Distributed Research Infrastructure
for Advanced Electron Microscopy. Microscopy
and Microanalysis.

