# Supplementary material for: Interfaces in Epitaxially Grown Zn3P2 Nanowires and Their Composition-Dependent Optoelectronic Properties for Photovoltaic Applications
Source: Chem Mater. 2025 Jul 21;37(15):5805–13. doi: 10.1021/acs.chemmater.5c00985 (PMC12355644; doi:10.1021/acs.chemmater.5c00985)
Supplement: Supplementary file 1 [file cm5c00985_si_001.pdf]

Supplementary information to "Interfaces in epitaxially grown Zn_3P_2 nanowires and their composition dependent optoelectronic properties for photovoltaic applications"

Simon Escobar Steinvall[1*], Francesco Salutari[2], Jonas Johansson[3], Ishka Das[4], Sebastian Lehmann[1], Stephen A. Church[4], Maria Chiara Spadaro[2,5], Patrick Parkinson[4], Jordi Arbiol[2,6], Kimberly A. Dick[1]

1. Center for Analysis and Synthesis and NanoLund, Lund University, Box 124, 221 00 Lund, Sweden
2. Catalan Institute of Nanoscience and Nanotechnology (ICN2), CSIC and BIST, 08193 Barcelona, Catalonia, Spain
3. Division of Solid State Physics and NanoLund, Lund University, 221 00 Lund, Sweden
4. Department of Physics and Astronomy and The Photon Science Institute, The University of Manchester, Manchester, M13 9PL United Kingdom
5. Department of Physics and Astronomy "Ettore Majorana", University of Catania and CNR-IMM Via S. Sofia 64, 95123 Catania, Italy
6. ICREA, 08010 Barcelona, Catalonia, Spain
*Corresponding Author: simon.escobar_steinvall@chem.lu.se

While not explicitly investigated, we did notice a history effect on the growth. At the start of a growth session, we always had a clean chamber and an InP base cover. With subsequent growth we did see the formation of a Zn-P cover in the chamber. There was an influence on the growth rate, mainly between the first growth and second growth where it increased, after which it quickly stabilises. However, the main influence was on the In nanoparticle formation. After some experiments we started to observe a decrease in the nanowire density, and eventually the nanowire formation was supressed completely. In short, the Zn-P cover interferes with the In nanoparticle deposition. We could return back to initial conditions by re-depositing our InP base cover, resetting any history effects.

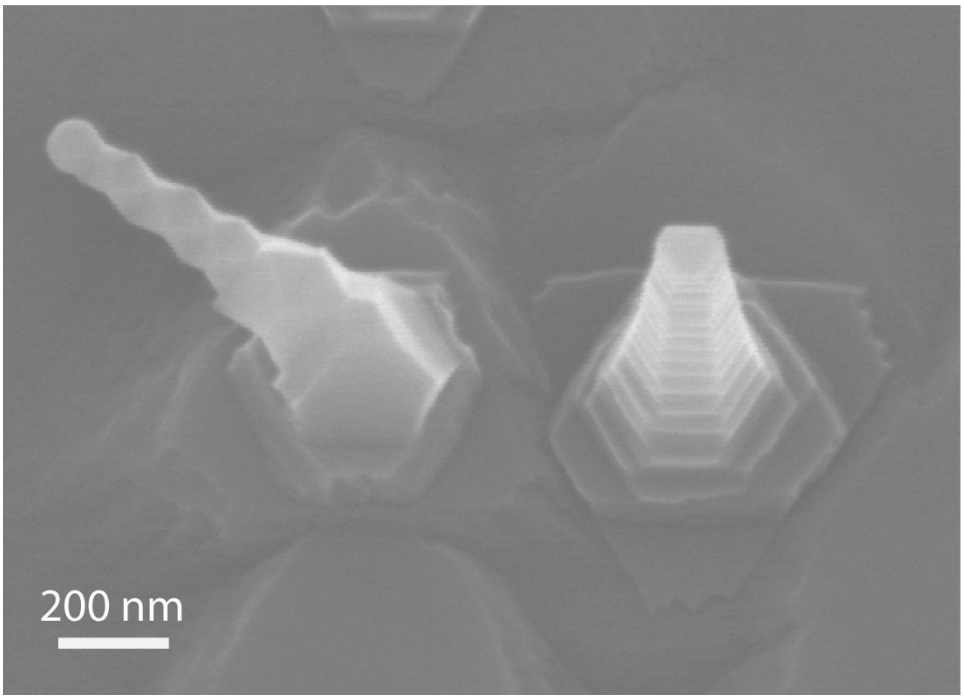

Figure S1. High magnification SEM image of nanowire at high V/II with and without out catalyst particles.

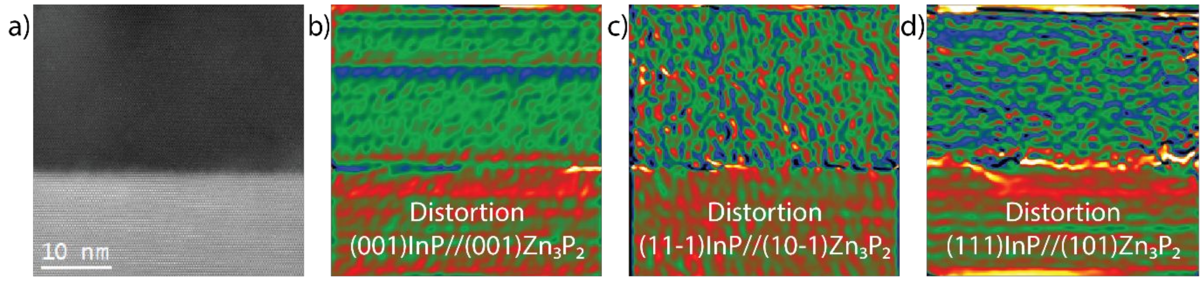

Figure S2. (a) AC-HAADF STEM image and (b-d) corresponding GPA maps of interface between InP (111)B and Zn_3P_2 nanowires showing no regular misfit dislocations. The blue horizontal line observed in the dilatation maps for the (001) planes correspond to a STEM scanning artifact, and not a defect in the material.

In Figure S3 we show a region at the base of the Zn_3P_2 nanowire grown on InP (111)A. By filtering the power spectrum of the HAADF-STEM images (Figure S3a-b) and taking the InP substrate as reference, we highlight the rotated region in green belonging to the wire (<100> zone axis) and the non-rotated region in red from the Zn_3P_2 layer in between the nanowires (<111> zone axis)(Figure S3c). By applying GPA focusing on the $(001)_{Zn_3P_2}$// $(001)_{InP}$, we clearly observe the formation of defects along the interface between the thin film and the substrate. These defects appear in correspondence with residual twinning and re-arrangement of the substrate as shown by overlapping the GPA dilatation map and original HAADF image (Figure S3d).

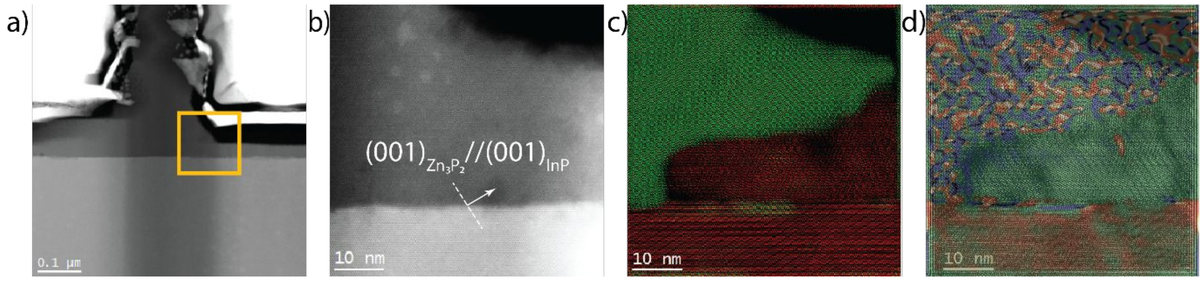

Figure S3. (a) Low-magnification HAADF-STEM image of the interface of a Zn_3P_2 nanowire/thin film and InP (111)A substrate. (b) High-magnification HAADF-STEM image of area indicated in (a). (c) Colour map showing the different crystal orientations based on filtered power spectrum, showing the areas with a <100> zone axis in green and <111> in red. (d) GPA dilatation map of the $(100)_{Zn3P2}$//$(001)_{InP}$ transposed on the HAADF-STEM image in (b).

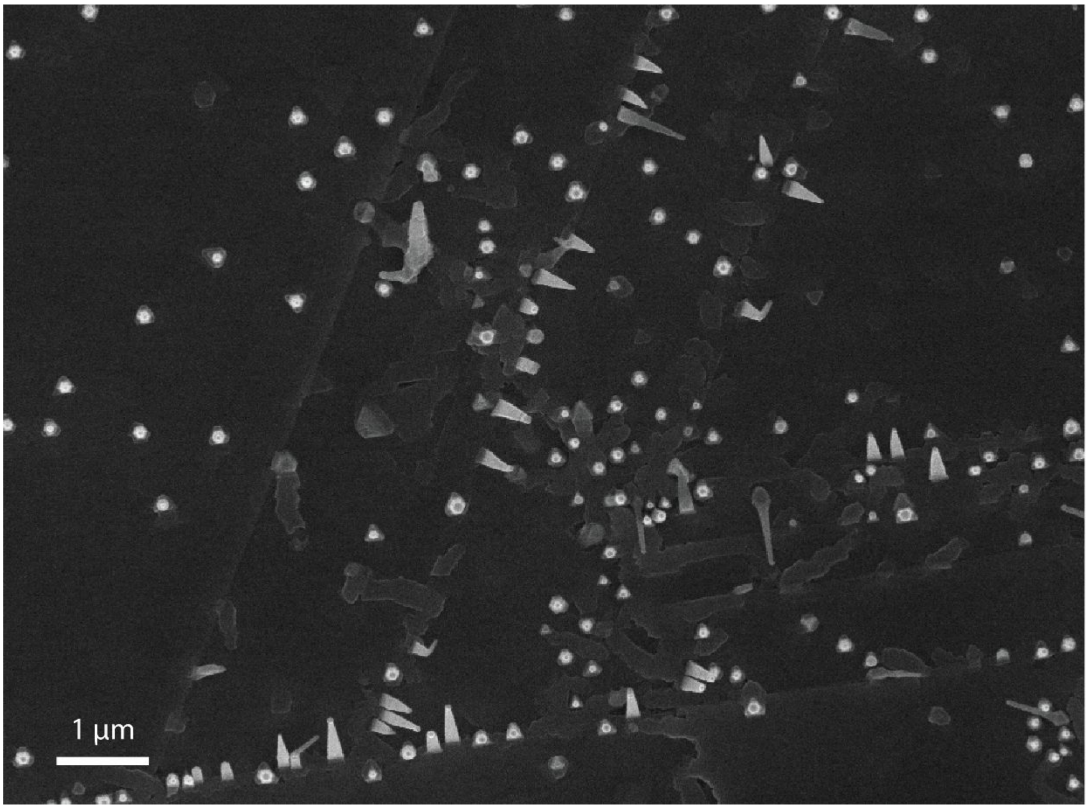

Figure S4. Top view SEM of tilted nanowires growing from surface defects.
